# Supplementary material for: Removing unwanted variation in a differential methylation analysis of Illumina HumanMethylation450 array data
Source: Nucleic Acids Res. 2015 May 18;43(16):e106. doi: 10.1093/nar/gkv526 (PMC4652745; doi:10.1093/nar/gkv526)
Supplement: SUPPLEMENTARY DATA [file supp_43_16_e106__index.html]

Removing unwanted variation in a differential methylation analysis of Illumina HumanMethylation450 array data — Removing unwanted variation in a differential methylation analysis of Illumina HumanMethylation450 array data — SUPPLEMENTARY DATA 

# Removing unwanted variation in a differential methylation analysis of Illumina HumanMethylation450 array data

## SUPPLEMENTARY DATA

- SUPPLEMENTARY DATA
- SUPPLEMENTARY DATA
- SUPPLEMENTARY DATA
- SUPPLEMENTARY DATA
- SUPPLEMENTARY DATA
- SUPPLEMENTARY DATA
- SUPPLEMENTARY DATA
- SUPPLEMENTARY DATA
- SUPPLEMENTARY DATA
