# Supplementary material for: Removing unwanted variation in a differential methylation analysis of Illumina HumanMethylation450 array data
Source: Nucleic Acids Res. 2015 May 18;43(16):e106. doi: 10.1093/nar/gkv526 (PMC4652745; doi:10.1093/nar/gkv526)
Supplement: SUPPLEMENTARY DATA [file supp_gkv526_nar-00167-met-n-2015-File008.docx]

SUPPLEMENTARY LEGENDS

Supplementary Table 1. Ageing samples used in this study.

Supplementary Table 2. Liu et al. (6) smoking samples used in this study.

Supplementary Table 3. Hannum et al. (45) and Liu et al. (6) samples used in this study to create the smoking+ dataset (Combination 1).

Supplementary Table 4. Hannum et al. (45) and Liu et al. (6) samples used in this study (Combination 2).

Supplementary Table 5. Hannum et al. (45) and Liu et al. (6) samples used in this study (Combination 3).

Supplementary Table 6. 187 smoking CpGs identified and replicated by Zeilinger et al. (46) used as true positives in this study.

Supplementary Table 7. TCGA LUAD samples used in this study.

Supplementary Table 7. TCGA LUAD samples used in this study.
