# Supplementary material for: Removing unwanted variation in a differential methylation analysis of Illumina HumanMethylation450 array data
Source: Nucleic Acids Res. 2015 May 18;43(16):e106. doi: 10.1093/nar/gkv526 (PMC4652745; doi:10.1093/nar/gkv526)
Supplement: SUPPLEMENTARY DATA [file supp_gkv526_nar-00167-met-n-2015-File009.pdf]

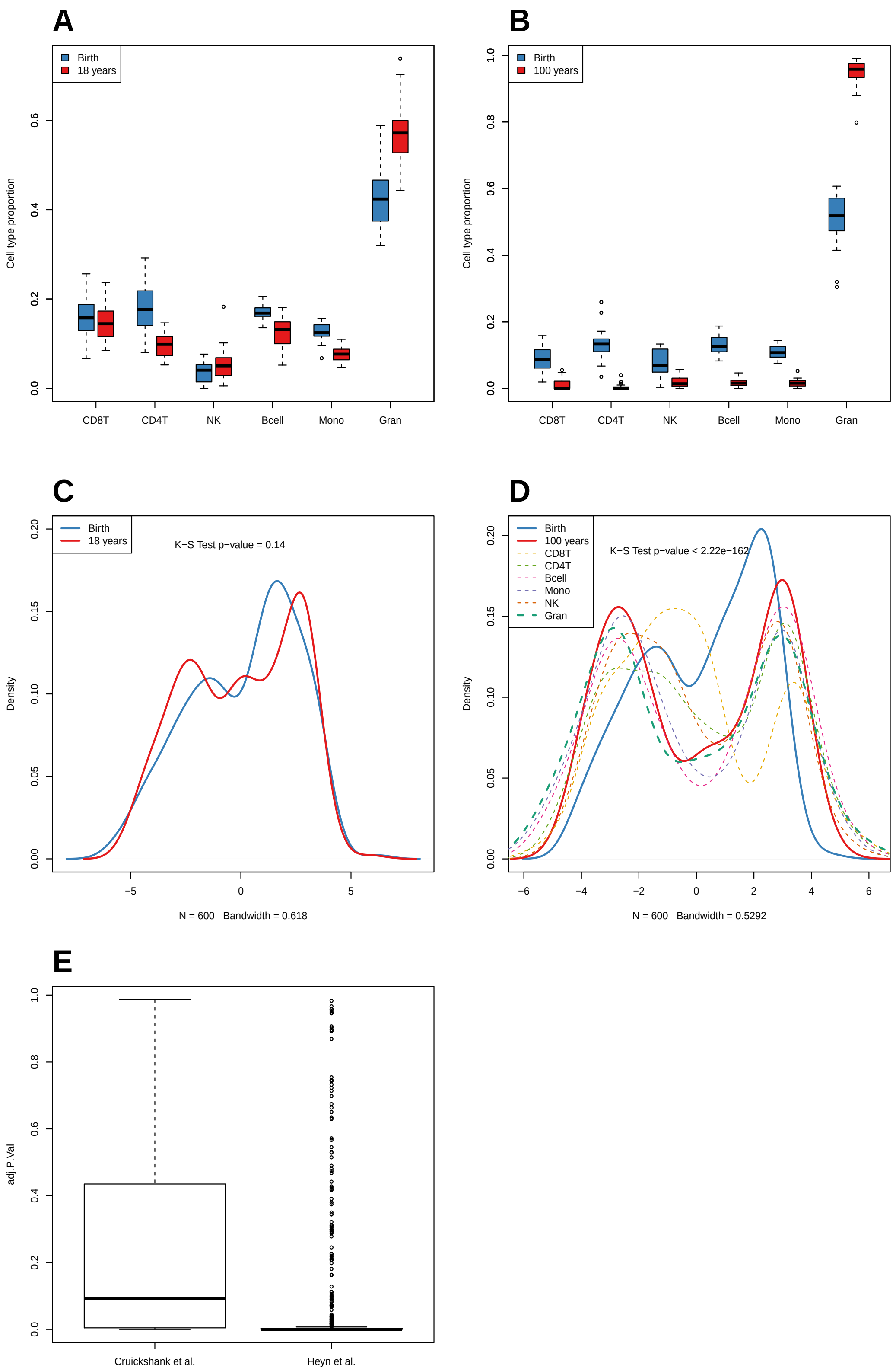

Supplementary Figure 1: Analysis of cell type composition in the ageing data truth set (Cruickshank et al.) and another ageing dataset known to have cell type composition issues (Heyn et al.). (A) Estimates of the proportions of various blood cell subtypes in the Cruickshank et al. ageing data. (B) Estimates of the proportions of various blood cell subtypes in the Heyn et al. ageing data. (C) Distribution of methylation M-values for the 600 cell type discriminating probes in the Cruickshank et al. data. (D) Distribution of methylation M-values for the 600 cell type discriminating probes in the Heyn et al. data. Also shows distributions for the same probes in sorted blood cell subtypes. (E) Distribution of FDR adjusted p-values for the DM analysis between birth and later time point of the 600 cell type discriminating probes in the Cruickshank and Heyn et al. datasets.

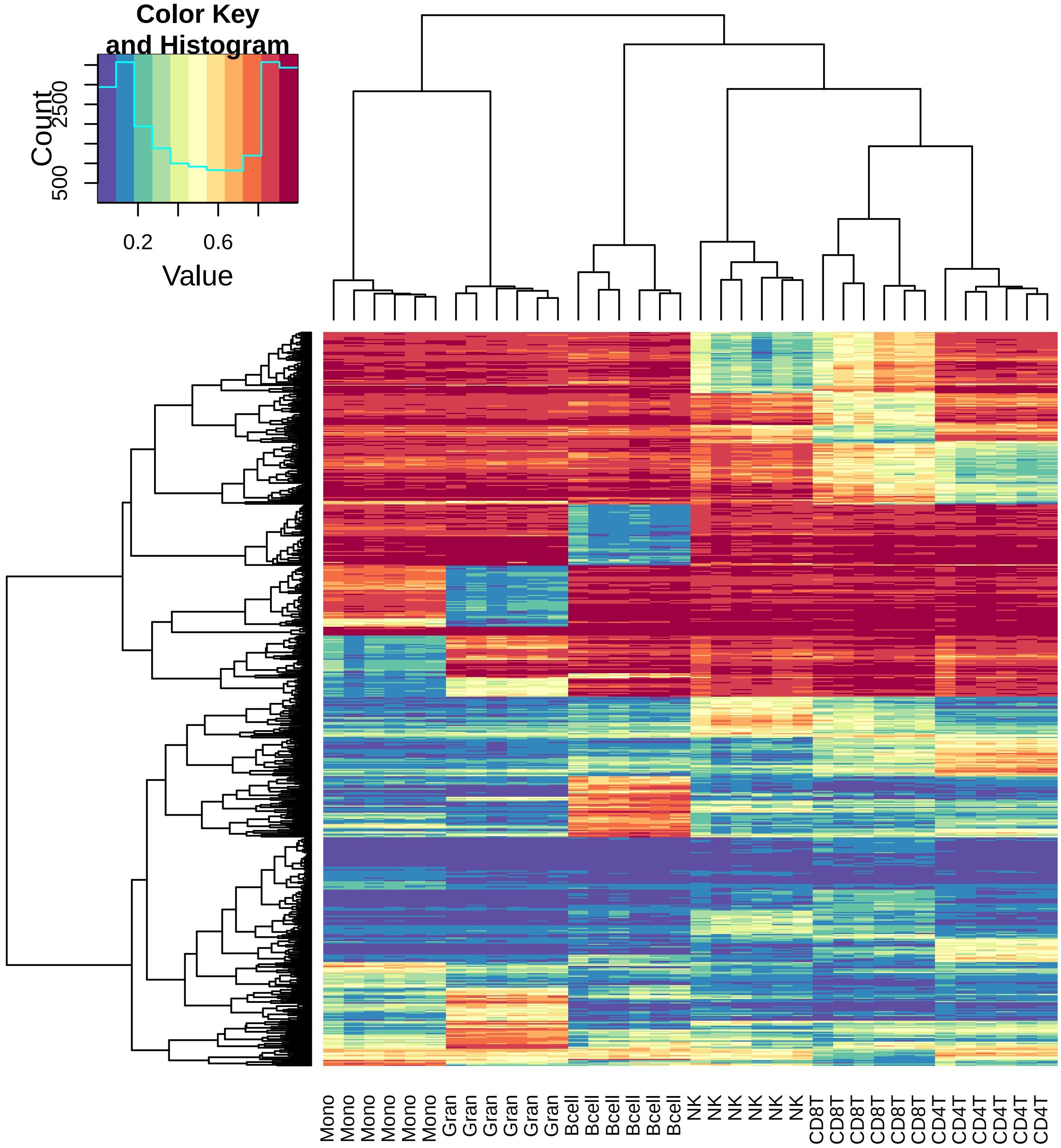

Supplementary Figure 2: Heat map of the 600 blood cell subtype discriminating probes identified from sorted blood cell data using the method described by Jaffe and Irizarry (2014).

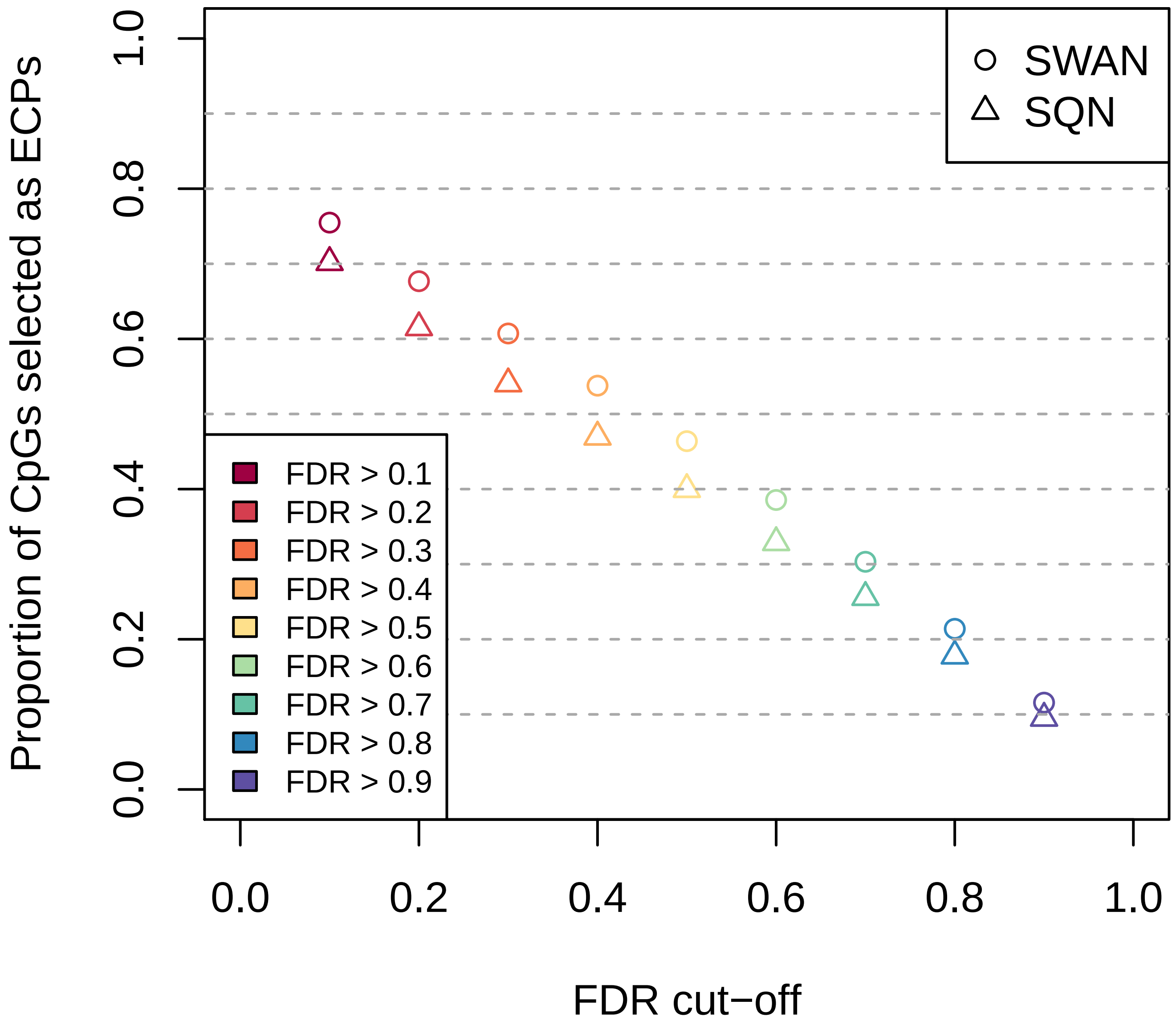

Supplementary Figure 3: The proportion of CpGs selected as ECPs at various FDR cut-offs following an initial DM analysis of the ageing+ data pre-processed using 2 different methods.

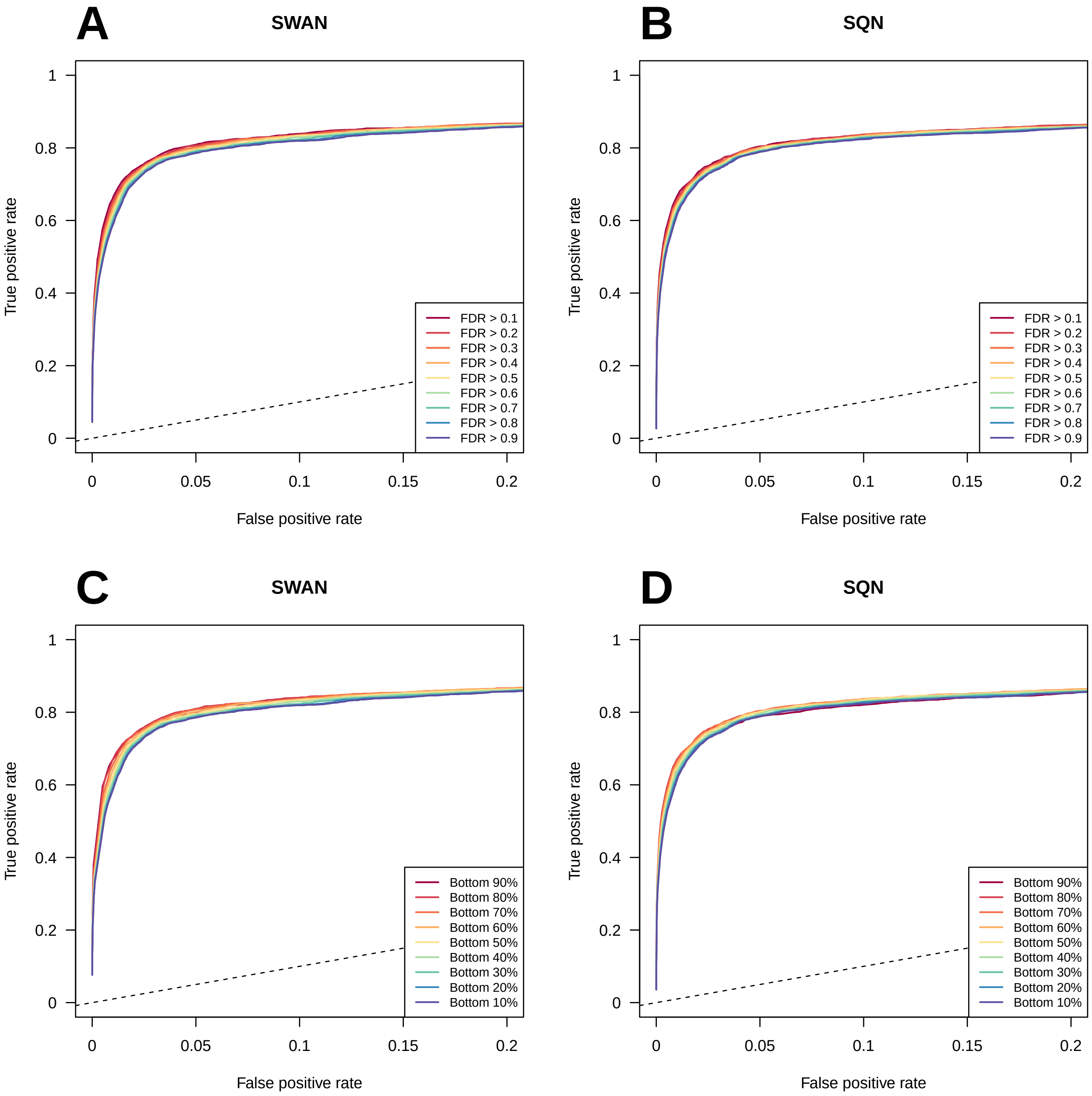

Supplementary Figure 4: ROC curves for the DM analysis of the ageing+ data using the 2-stage RUVm approach exploring the effect on performance of ECP selection. (A) ECPs are selected based on FDR cut-off and data is pre-processed using SWAN. (B) ECPs are selected based on FDR cut-off and data is pre-processed using SQN. (C) ECPs are selected based on taking a fixed percentage from the bottom of the ranked list and data is pre-processed using SWAN. (D) ECPs are selected based on taking a fixed percentage from the bottom of the ranked list and data is pre-processed using SQN.

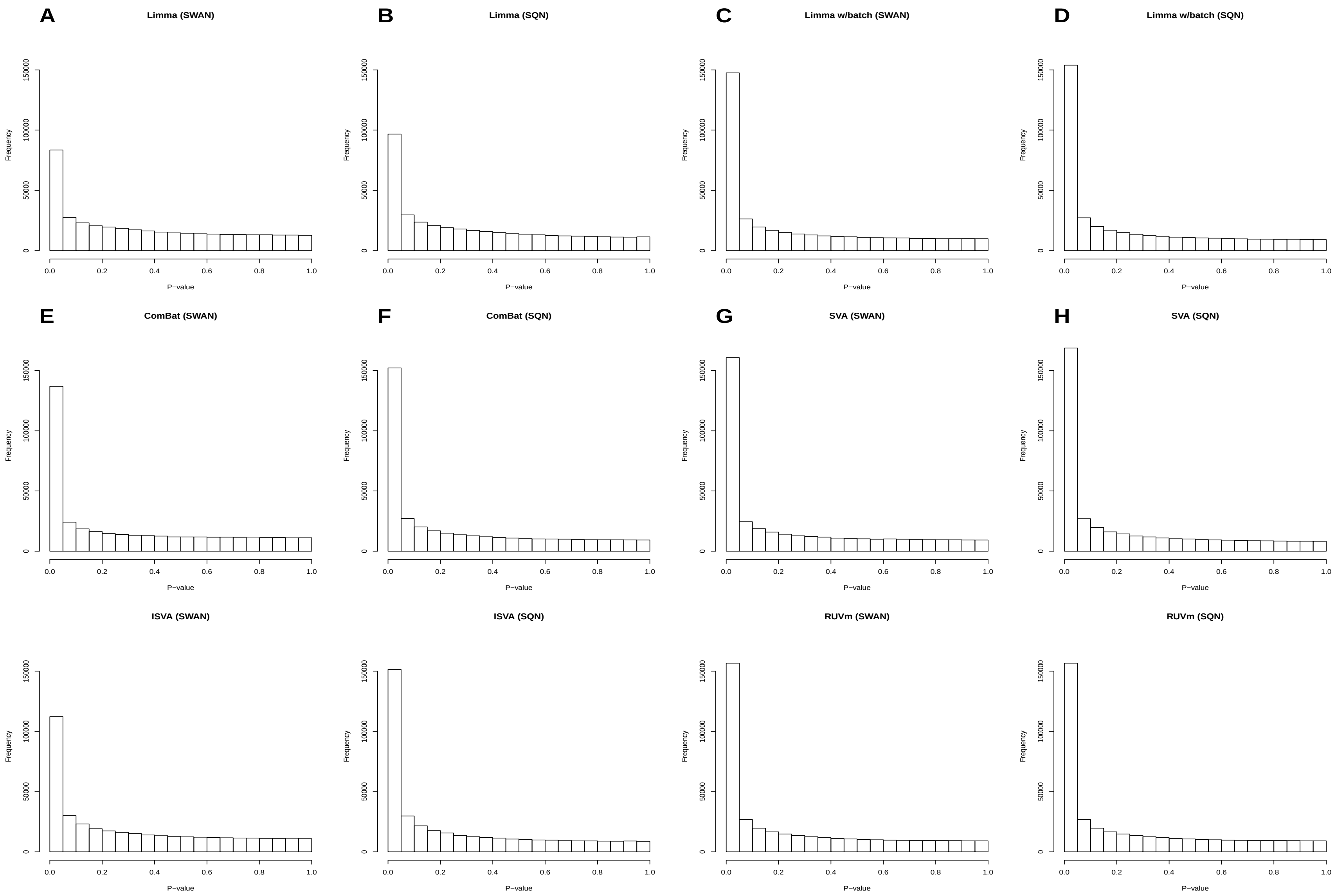

Supplementary Figure 5: P-value histograms for the DM analysis of the ageing+ data using various methods. (A) Limma (SWAN). (B) Limma (SQN). (C) Limma with factor for study (SWAN). (D) Limma with factor for study (SQN). (E) ComBat (SWAN). (F) ComBat (SQN).(G) SVA (SWAN). (H) SVA (SQN). (I) ISVA (SWAN). (J) ISVA (SQN). (K) RUVm (SWAN). (L) RUVm (SQN).

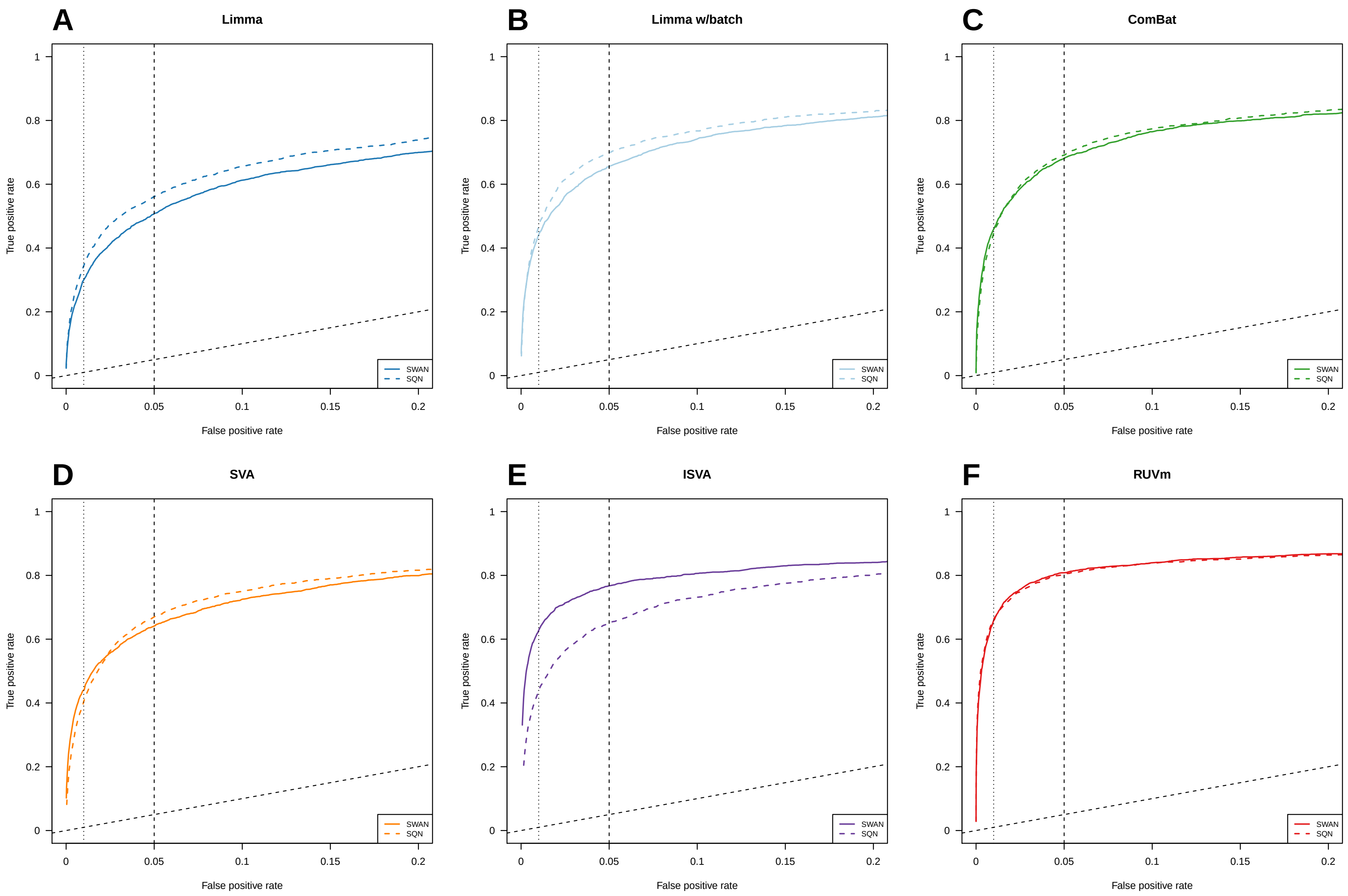

Supplementary Figure 6: ROC curves for the DM analysis of the ageing+ data using the 2-stage RUVm approach exploring the effect of pre-processing on the performance of various methods. (A) Limma (B) Limma with factor for study (C) ComBat (D) SVA (E) ISVA (F) RUVm.

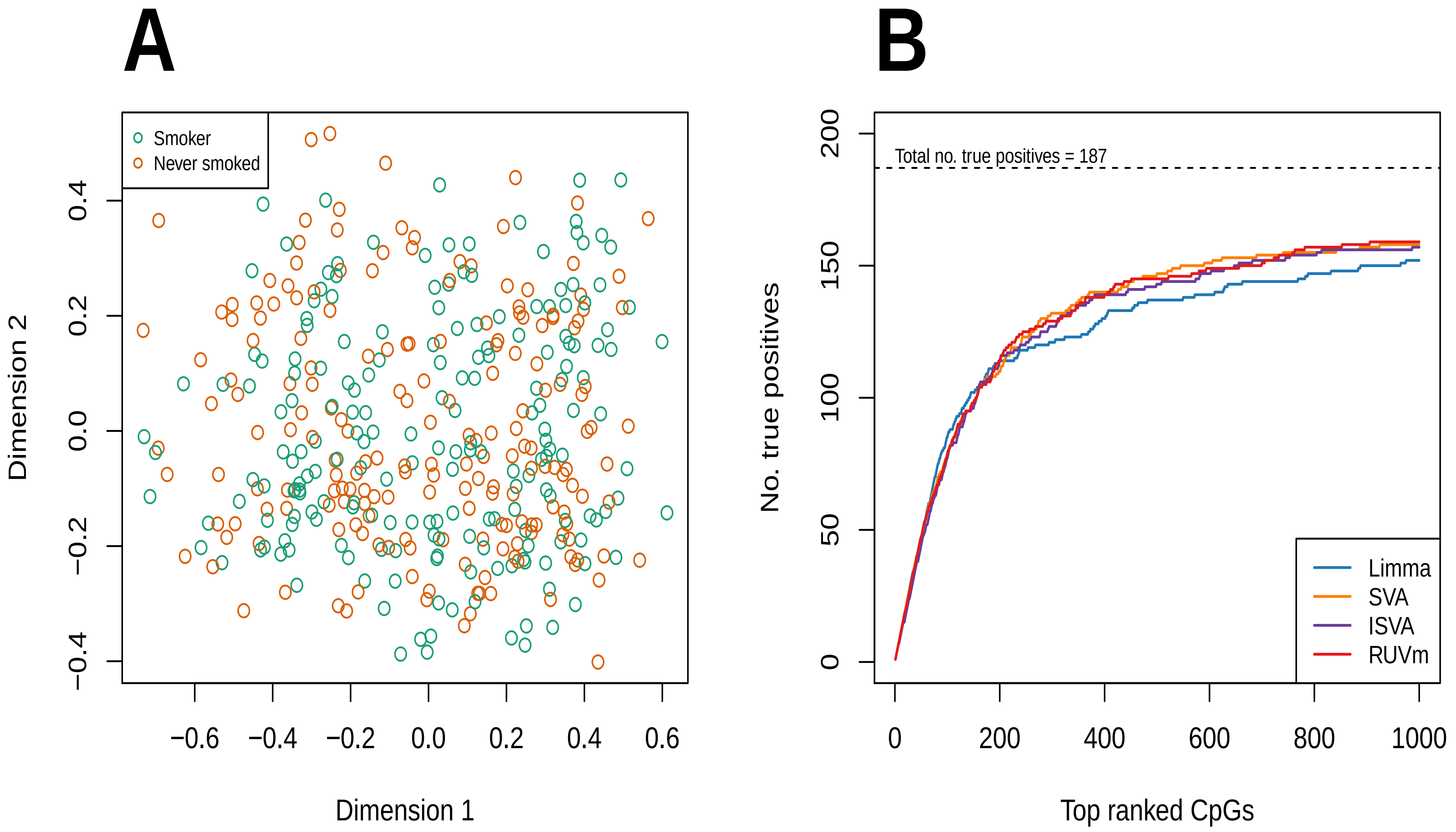

Supplementary Figure 7: DM analysis of smoking data. (A) MDS plot of 450k methylation data from 200 current and 193 never smokers. (B) Performance of various methods in a DM analysis of the smoking data: cumulative number of true positives ranked in the top 1000 DM CpGs by various analysis methods.

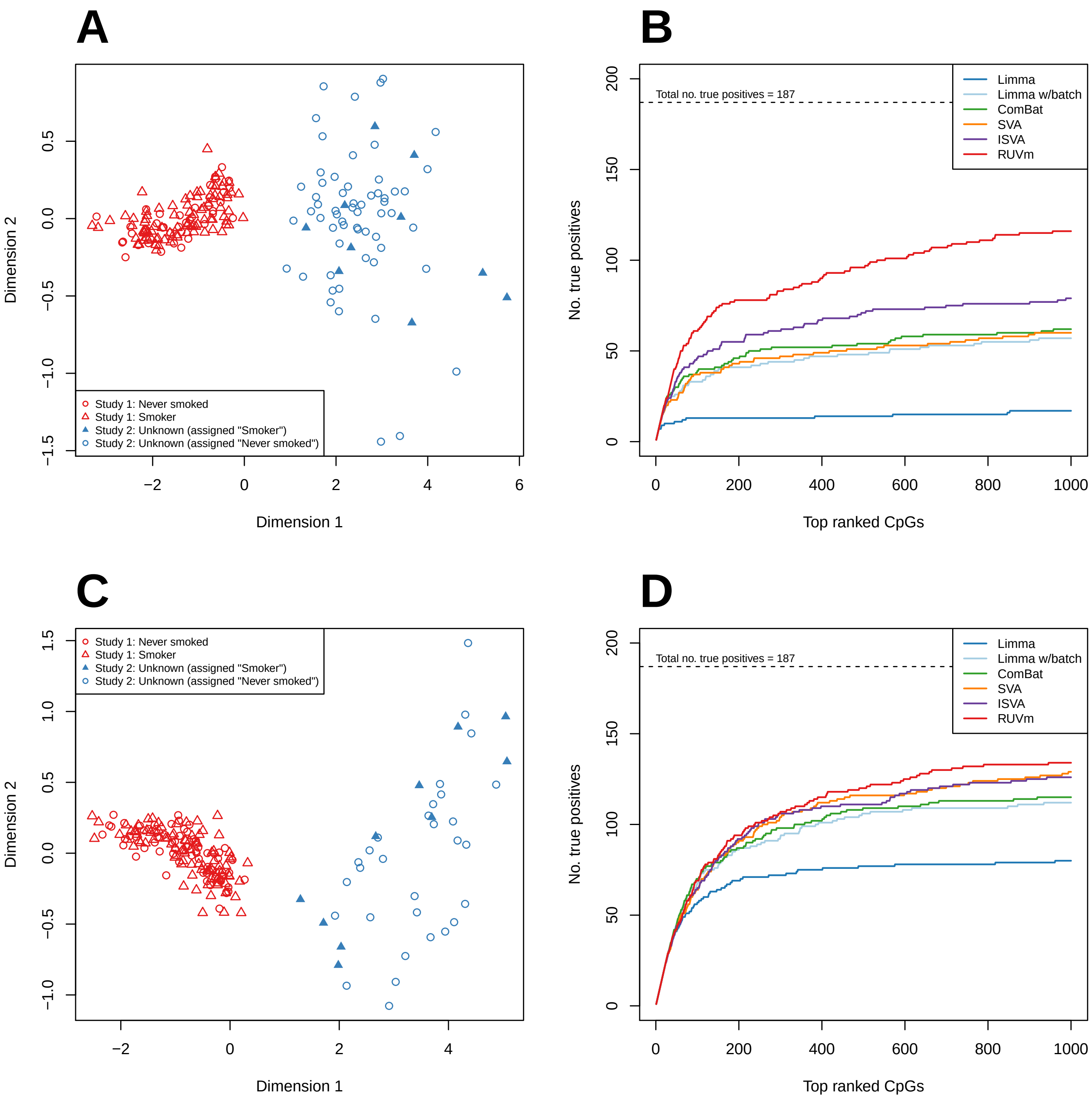

Supplementary Figure 8: DM analysis of two different combinations of samples from the Liu and Hannum "smoking" datasets. (A) MDS plot of a combination of 90 smokers and 40 never smokers from the Liu data and 70 samples from the Hannum data, 10 of which were assigned as "smokers" and 60 as "never" smokers. (B) Performance of various analysis methods in a DM analysis of the data described in (A). The lines represent the cumulative number of true positives ranked in the top 1000 CpGs produced by the different methods. (C) MDS plot of a combination of 90 smokers and 70 never smokers from the Liu data and 40 samples from the Hannum data, 10 of which were assigned as "smokers" and 30 as "never" smokers. (D) Performance of various analysis methods in a DM analysis of the data described in (C). The lines represent the cumulative number of true positives ranked in the top 1000 CpGs produced by the different methods.

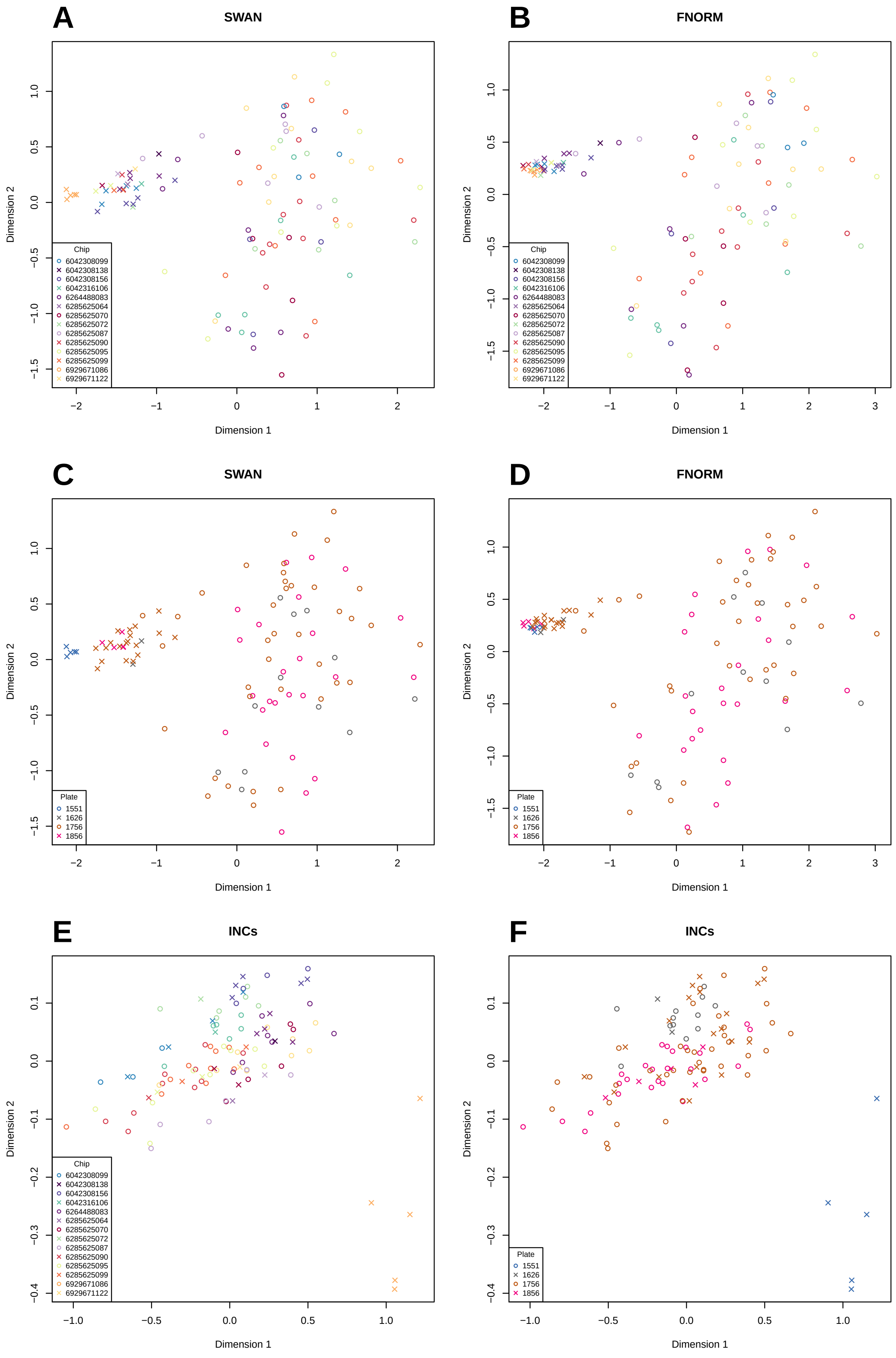

Supplementary Figure 9: MDS plots of the 450k LUAD data coloured by chip (A, B) and plate (C, D). MDS plot of the LUAD INCs coloured by chip (E) and plate (F).

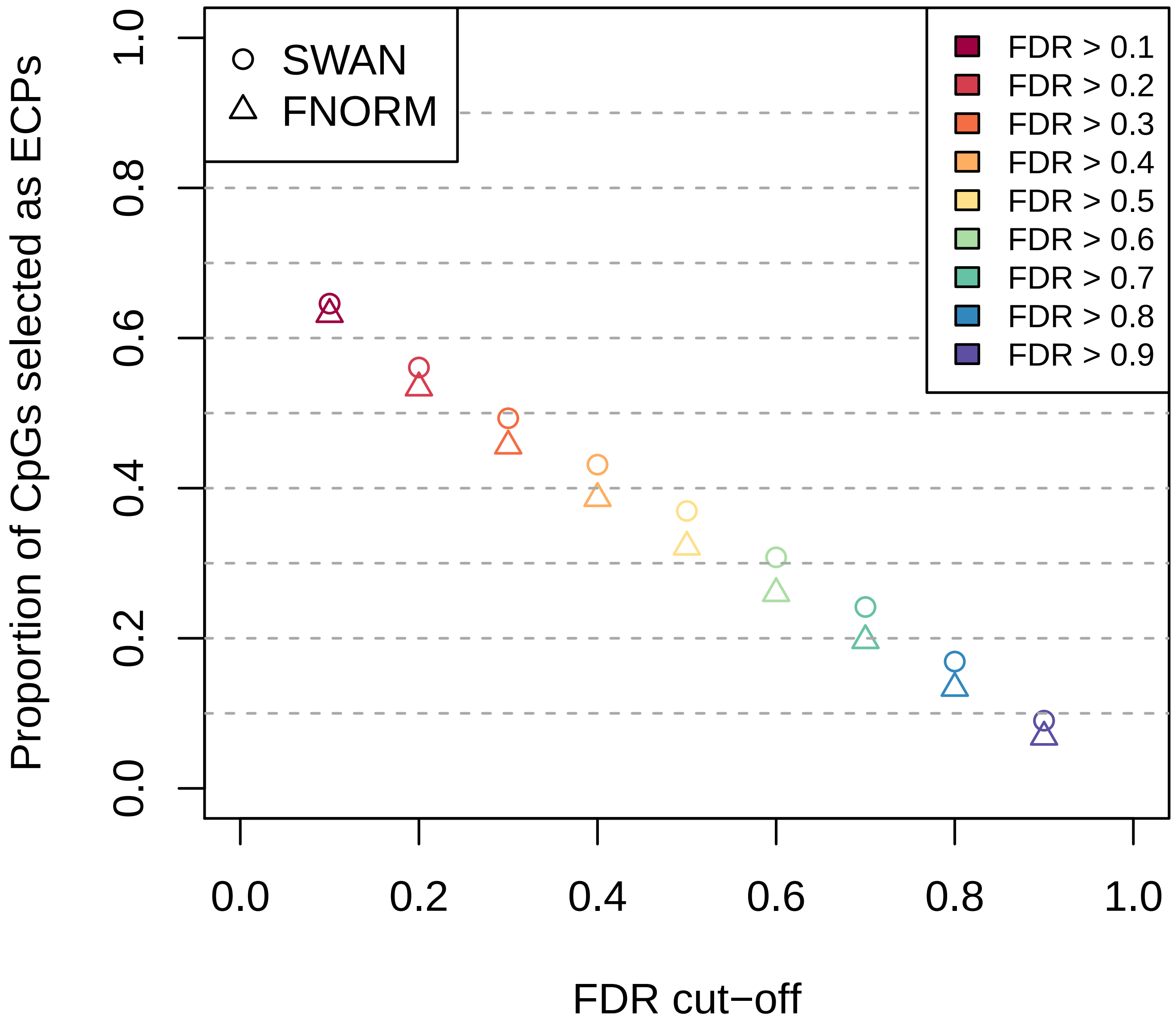

Supplementary Figure 10: The proportion of CpGs selected as ECPs at various FDR cut-offs following an initial DM analysis of the 450k TCGA LUAD data (31 normal, 75 tumour samples) pre-processed using 2 different methods.

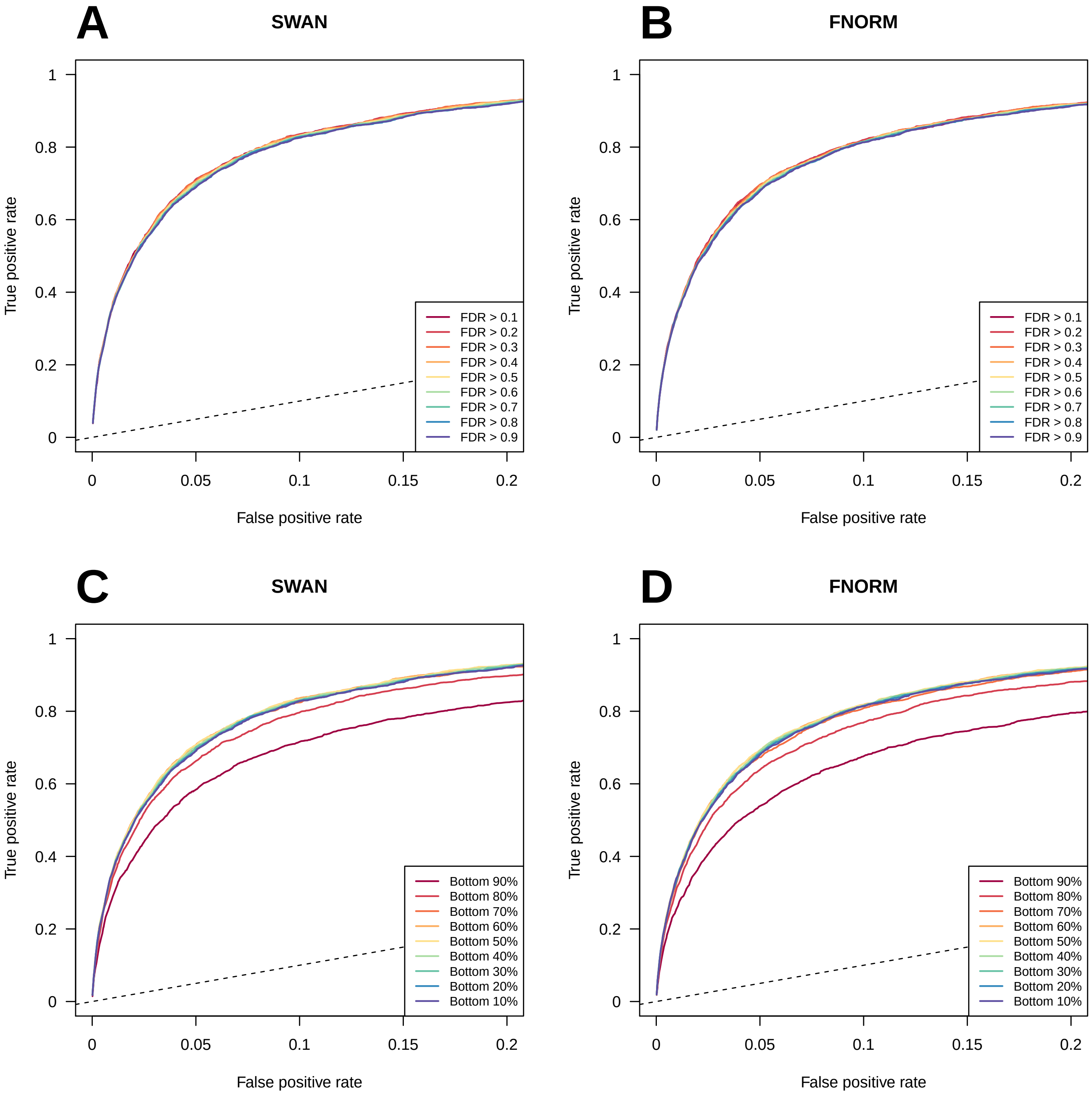

Supplementary Figure 11: ROC curves for the DM analysis of the 450k TCGA LUAD data (31 normal, 75 tumour samples) using the 2-stage RUVm approach exploring the effect on performance of ECP selection. (A) ECPs are selected based on FDR cut-off and data is pre-processed using SWAN. (B) ECPs are selected based on FDR cut-off and data is pre-processed using FNORM. (C) ECPs are selected based on taking a fixed percentage from the bottom of the ranked list and data is pre-processed using SWAN. (D) ECPs are selected based on taking a fixed percentage from the bottom of the ranked list and data is pre-processed using FNORM.

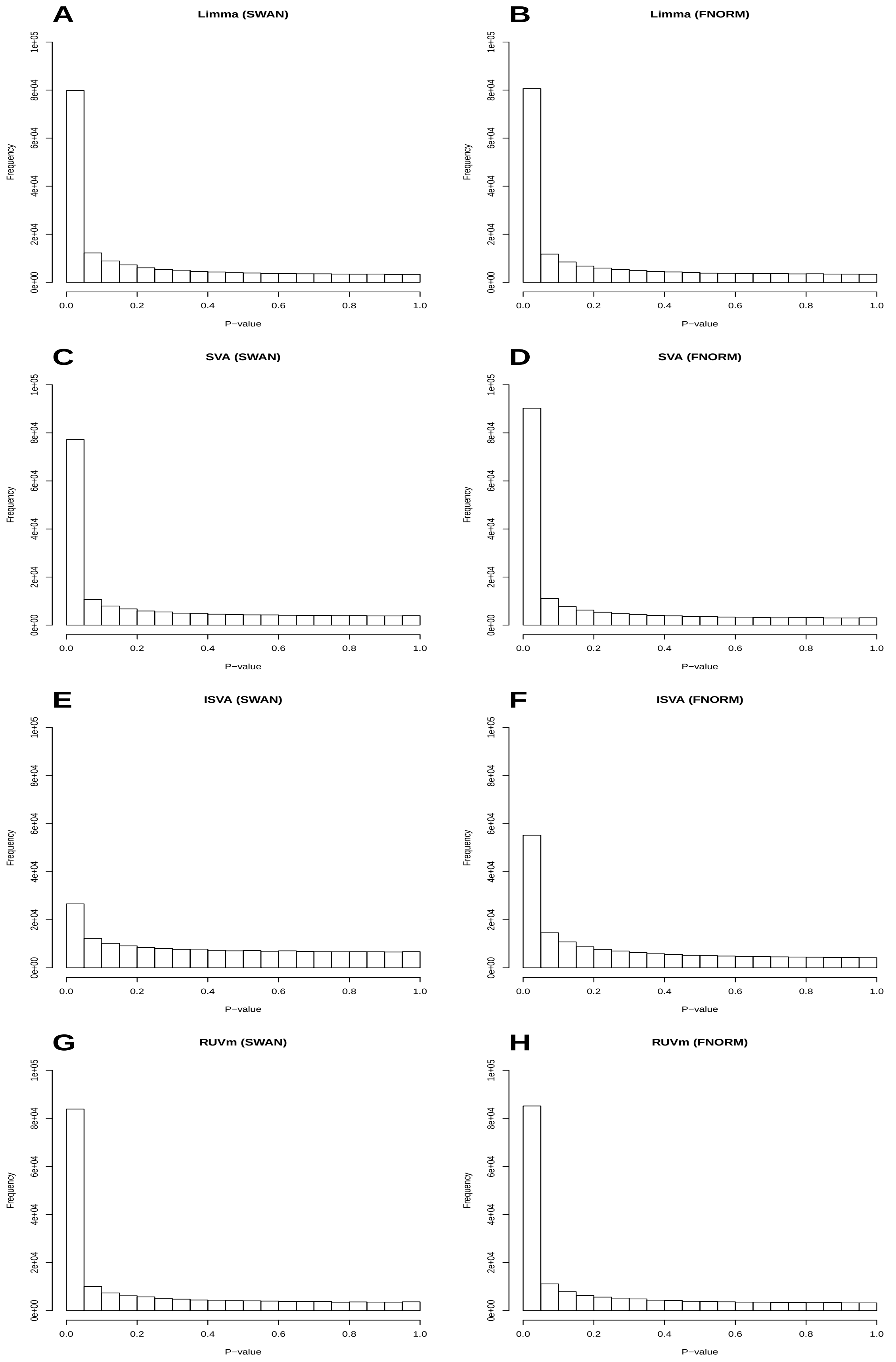

Supplementary Figure 12: P-value histograms for the DM analysis of the 50k TCGA LUAD data (31 normal, 75 tumour samples) using various methods. (A) Limma (SWAN). (B) Limma (FNORM). (C) SVA (SWAN). (D) SVA (FNORM). (E) ISVA (SWAN). (F) ISVA (FNORM). (G) RUVm (SWAN). (H) RUVm (FNORM).

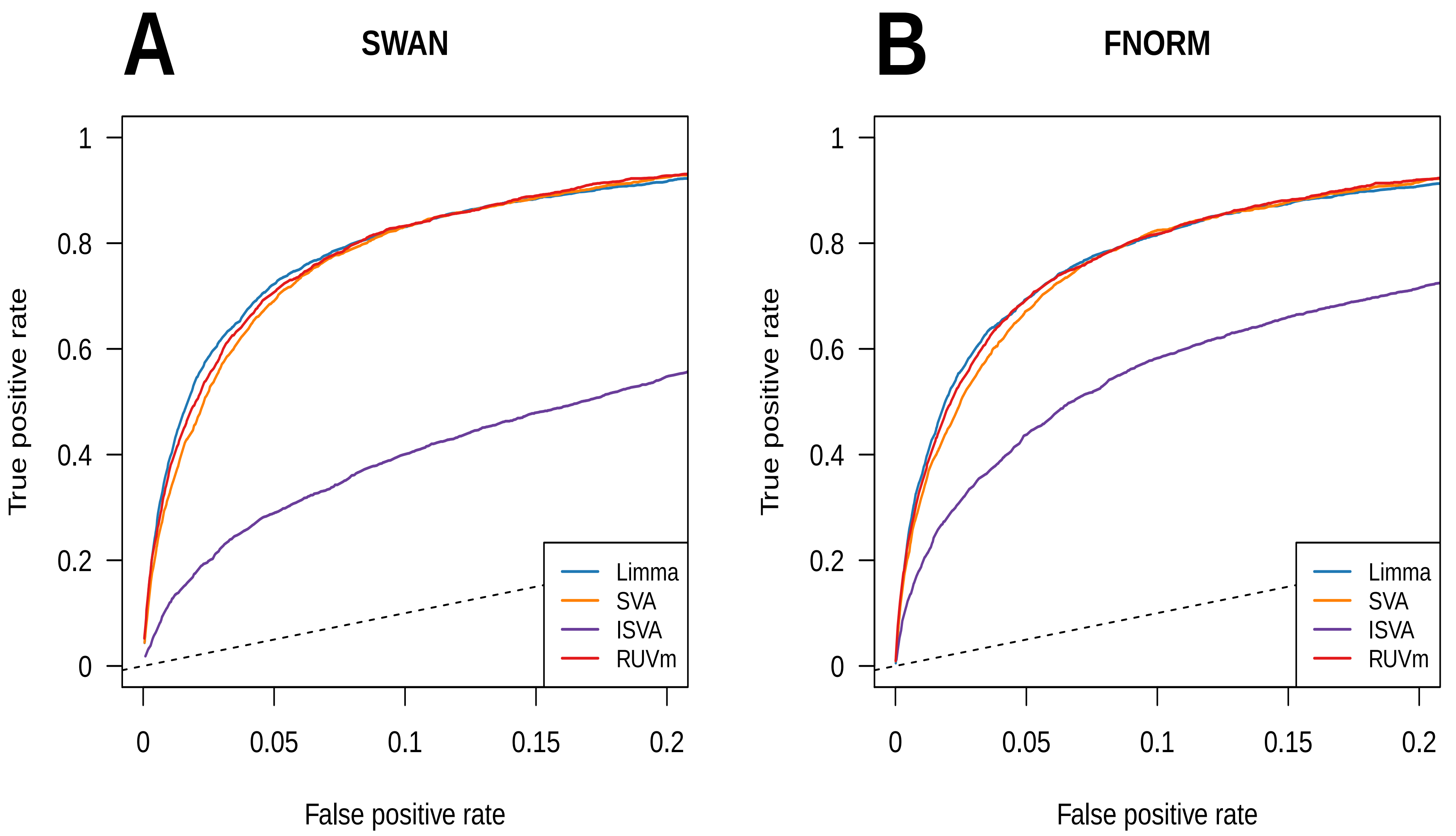

Supplementary Figure 13: Performance of various adjustment methods in a DM analysis of the TCGA LUAD data. (A) ROC curve showing the false positive rate versus the true positive rate for the various adjustment methods on the SWAN pre-processed data. (B) ROC curve showing the false positive rate versus the true positive rate for the various adjustment methods on the FNORM pre-processed data.

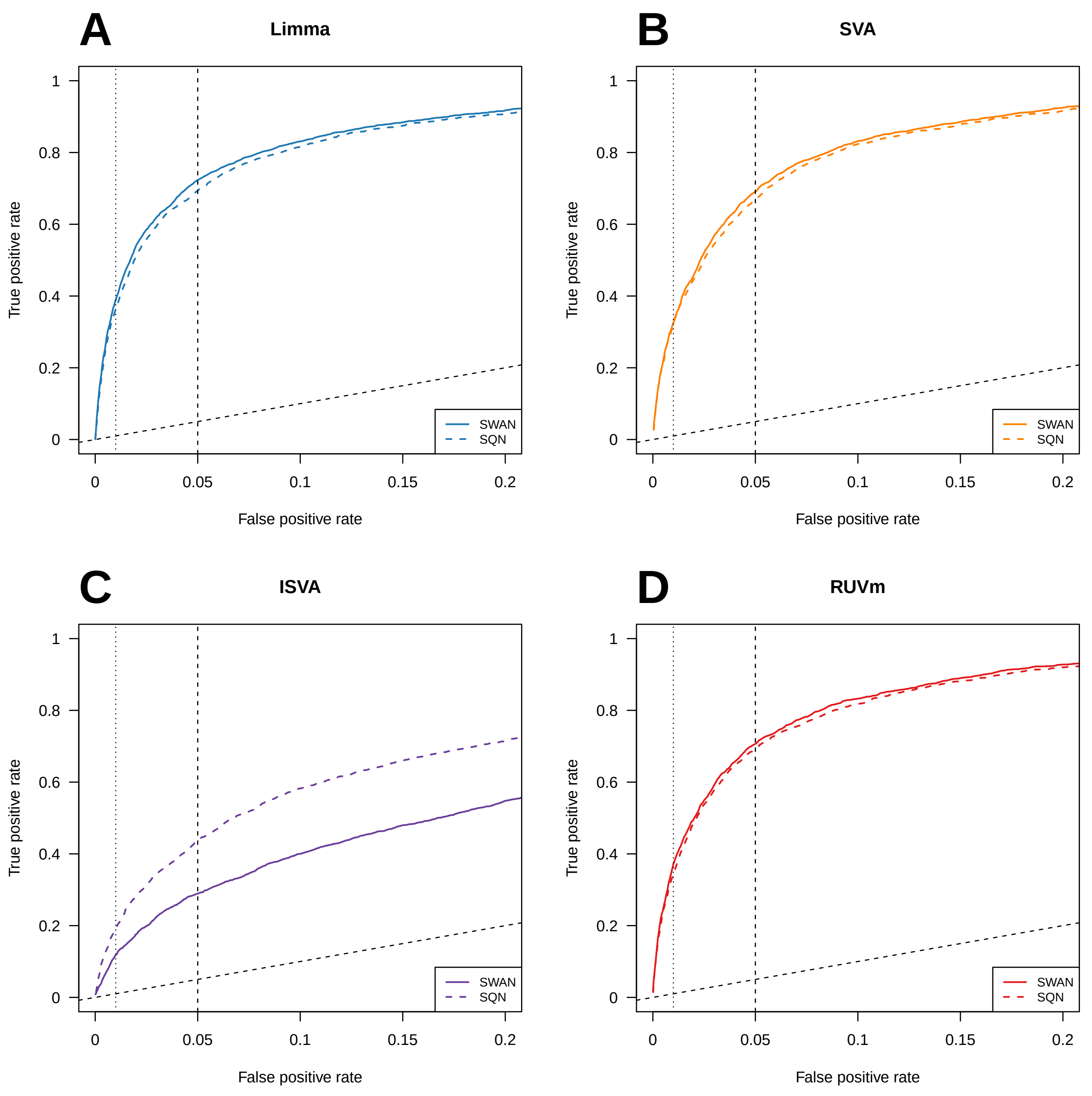

Supplementary Figure 14: ROC curves for the DM analysis of the LUAD data using the 2-stage RUVm approach exploring the effect of pre-processing on the performance of various methods. (A) Limma (B) SVA (C) ISVA (D) RUVm.

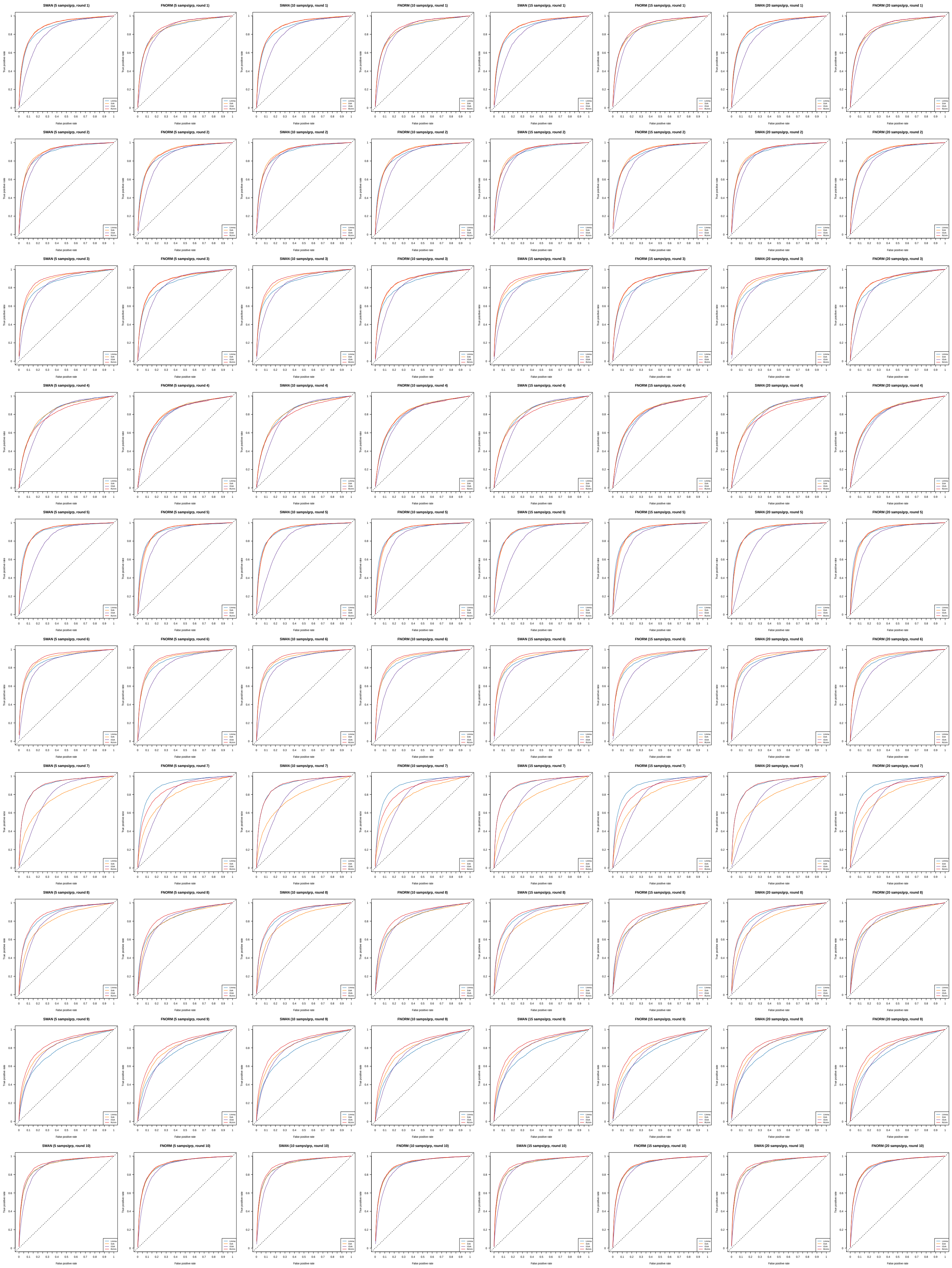

Supplementary Figure 15: ROC curves for the DM analysis of the 450k TCGA LUAD data using various methods at different levels of subsampling.

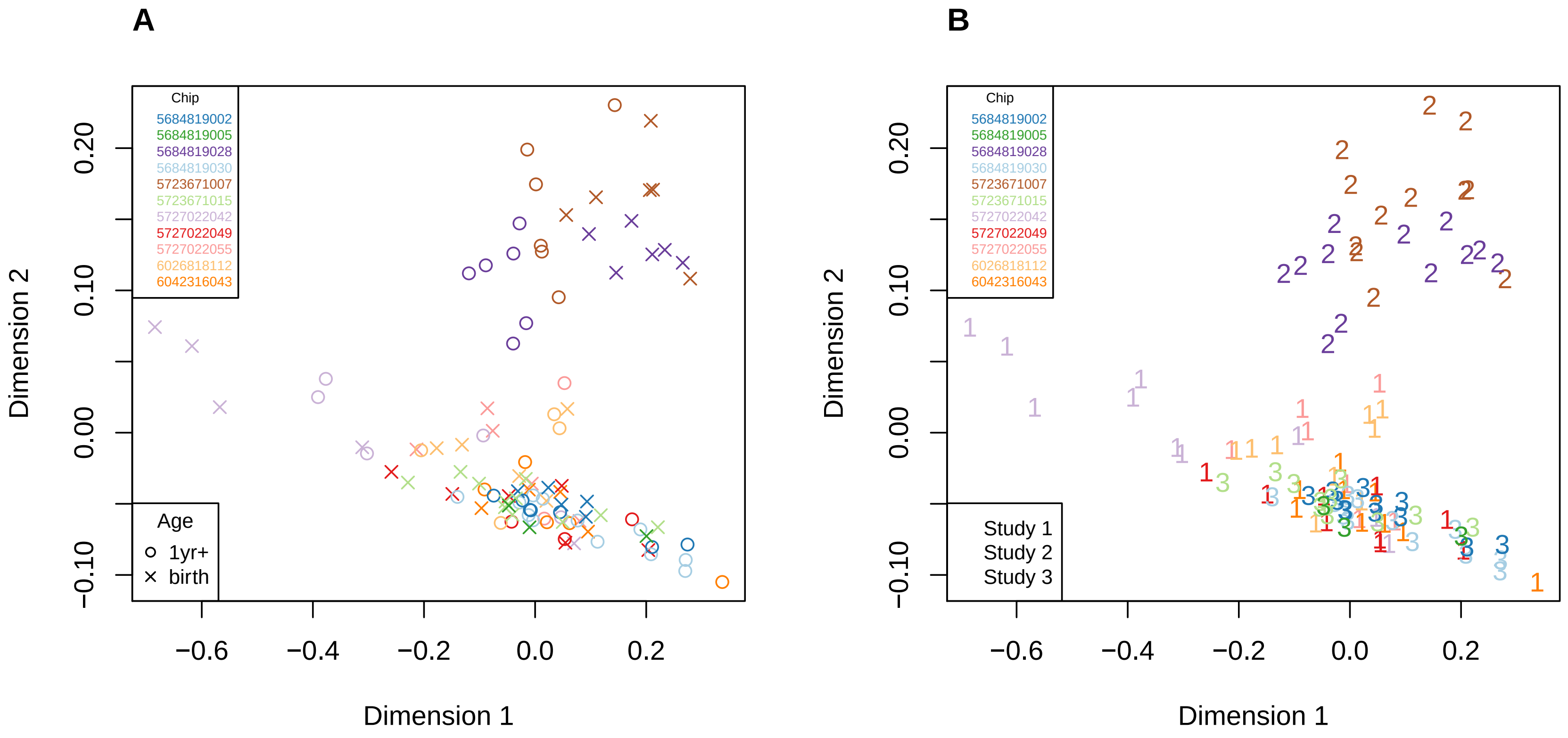

Supplementary Figure 16: MDS plots of the INCs associated with the ageing+ data coloured by chip.
