# Supplementary material for: Removing unwanted variation in a differential methylation analysis of Illumina HumanMethylation450 array data
Source: Nucleic Acids Res. 2015 May 18;43(16):e106. doi: 10.1093/nar/gkv526 (PMC4652745; doi:10.1093/nar/gkv526)
Supplement: SUPPLEMENTARY DATA [file supp_gkv526_nar-00167-met-n-2015-File010.pdf]

Supplementary Methods: RUV-inverse

Assume there are m arrays and n CpGs. Let Y be an $m \times n$ matrix such that Y_{ij} is the M-value for the jth CpG on the ith array. We model Y as

$$Y_{m\times n} = X_{m\times p}\beta_{p\times n} + W_{m\times k}\alpha_{k\times n} + \epsilon_{m\times n} \tag{1}$$

where X is a matrix of biological factors of interest and W is a matrix of unknown, unwanted factors. Optionally, we may also wish to include an additional $Z\gamma$ term in the model, where Z is a matrix of known covariates; see [?] for details. We assume that $\text{Rank}\,[(X \mid W)] = p + k < m$.

We assume that X, W, and β are fixed. We assume that α and ϵ are random. The stochastic assumptions on α and ϵ are:

$$\begin{aligned}
\epsilon_{ij} &\sim N(0, \sigma_j^2) && (2)\\
\alpha_{ij} &\sim N(0, 1) && (3)\\
\alpha &\perp\!\!\!\perp \epsilon && (4)\\
\epsilon_{ij} &\perp\!\!\!\perp \epsilon_{i'j'} \text{ if } (i,j) \neq (i',j') && (5)\\
\alpha_{ij} &\perp\!\!\!\perp \alpha_{i'j'} \text{ if } (i,j) \neq (i',j') && (6)
\end{aligned}$$

Note in particular that the variance of ϵ_{ij} is allowed to differ for every CpG.

Let n_c denote the number of negative controls. Let Y_c denote the $m \times n_c$ submatrix of Y containing only the columns of the negative controls. Define β_c, α_c, and ϵ_c similarly. Assume that $\beta_c = 0$; this is the "negative control" assumption. It follows that

$$Y_c = W\alpha_c + \epsilon_c. \tag{7}$$

Define

$$G \equiv \frac{1}{n_c} Y_c Y_c'$$

and note that

$$\mathbb{E}\,[G] = WW' + \bar{\sigma}_c^2 I$$

where

$$\bar{\sigma}_c^2 \equiv \frac{1}{n_c} \sum_{j_c} \sigma_{j_c}^2.$$

Here j_c is an index variable that ranges over the indices of all of the negative controls. In words, $\bar{\sigma}_c^2$ is the average variance of the error terms of the negative controls.

Let Y_j denote the jth column of Y and note that

$$\text{Var}\,[Y_j] = WW' + \sigma_j^2 I.$$

In practice, we do not expect expect the σ_j^2 to vary too greatly from CpG to CpG, and we therefore assume that for all j, σ_j^2 is approximately equal to $\bar{\sigma}_c^2$, at least roughly. We may therefore consider G to be a rough approximation of $\text{Var}\,[Y_j]$. See [?] for a more detailed discussion of this point.

We may now define the RUV-inverse estimator for β. We define $\hat{\beta}$ as

$$\hat{\beta} \equiv \left[X'G^{-1}X\right]^{-1} X'G^{-1}Y. \tag{8}$$

We observe that this is essentially a feasible generalized least squares (FGLS) estimator.

We calculate the standard errors using the inverse method, as described in [X]. We briefly summarize the method here. The basic idea is to re-write (??) as

$$Y = X^\star\beta^\star + X\beta + W\alpha + \epsilon \tag{9}$$

where $X^\star$ is an $m \times 1$ matrix whose entries have been independently randomly generated following a standard normal distribution, and where $\beta^\star$ is $1 \times n$ matrix whose entires are all 0. We then fit the model, and calculate $\hat{\beta}^\star$.

The variance of $\hat{\beta}^\star_j$ (conditional on $X^\star$) can be well-approximated by a known, linear function of σ^2_j. By inverting this function, σ^2_j can be estimated as a function of $\hat{\beta}^\star_j$. (This inversion is where the inverse method gets its name.) The estimate of σ^2_j obtained in this way will be very noisy, because it is obtained using only one degree of freedom. However, by generating many different $X^\star$, repeating the process many times, and averaging the resulting estimates of σ^2_j, we obtain a much less noisy final estimate of σ^2_j. Once we have this estimate of σ^2_j, we may then use it to calculate the variance of $\hat{\beta}_j$ (conditional on X), and thus the standard errors.

It is possible to work through this process analytically, so that it is actually not necessary to generate random $X^\star$ and fit the resulting models. Again, see [?] for the details. Here we simply state the result, which can be expressed as a four-step procedure:

(1) Regress Y_c on X. Let R denote the residuals, i.e. $R \equiv Y_c - X(X'X)^{-1}X'Y_c$.

(2) Let UDU' be the eigendecomposition of RR'. Let d_i be the ith diagonal entry of D.

(3) Let $E_{m \times m}$ be a diagonal matrix with diagonal entries

$$e_i \equiv \begin{cases} \displaystyle\int_0^\infty \frac{dt}{d_i^2\,(1+2t/d_i^2)\prod_s^{m-p}\sqrt{1+2t/d_s^2}} & \text{if } 1 \le i \le m-p \\ 0 & \text{if } m-p < i \le m \end{cases}$$

(4) Let $\hat{\sigma}^2_j \equiv Y_j'UEU'Y_j$

References

[1] Gagnon-Bartsch, J.A., Jacob, L. and Speed, T.P. (2013) *Removing Unwanted Variation from High Dimensional Data with Negative Controls.* Tech. Rep. 820, Department of Statistics, University of California, Berkeley (2013).
